# Hematologic toxicity in *BRCA1* and *BRCA2* mutation carriers during chemotherapy: A retrospective matched cohort study

**DOI:** 10.1002/cam4.2471

**Published:** 2019-08-12

**Authors:** Allison H. West, Hayley Knollman, James Dugan, Donald Hedeker, Elisabeth A. Handorf, Sarah M. Nielsen, Lisa C. Bealin, Lindsay G. Goldblatt, Heather Willems, Mary B. Daly, Anosheh Afghahi, Olufunmilayo I. Olopade, Peter J. Hulick, Elena Shagisultanova, Dezheng Huo, Elias Obeid, Jane E. Churpek

**Affiliations:** ^1^ Department of Medicine Center for Clinical Cancer Genetics & Global Health University of Chicago Chicago IL USA; ^2^ Department of Medicine University of Chicago Chicago IL USA; ^3^ Risk Assessment Program Fox Chase Cancer Center Philadelphia PA USA; ^4^ Division of Medical Oncology University of Colorado—Denver Aurora CO USA; ^5^ Department of Public Health Sciences University of Chicago Chicago IL USA; ^6^ Department of Biostatistics Fox Chase Cancer Center Philadelphia PA USA; ^7^ Center for Medical Genetics NorthShore University HealthSystem Evanston IL USA; ^8^ Present address: Center for Medical Genetics NorthShore University HealthSystem Evanston IL USA; ^9^ Present address: Breast Cancer Program University of Pennsylvania Philadelphia PA USA

**Keywords:** *BRCA1*, *BRCA2*, breast cancer, hematologic toxicity, hereditary breast and ovarian cancer syndrome, neutropenic fever

## Abstract

Women who inherit a *BRCA1* or *BRCA2* mutation have an increased risk of breast cancer. Preliminary evidence suggests they may also have defects in bone marrow function. To test this hypothesis, we conducted a multicenter, retrospective, matched cohort study, comparing women with localized breast cancer requiring cytotoxic chemotherapy who carried an inherited *BRCA1* or *BRCA2* mutation to similar wild‐type patients treated between 1995 and 2017 and matched based on age, race, site, and chemotherapy regimen. The proportion who developed specific hematologic toxicities, timing of these toxicities, and patterns of blood count fluctuations over time were compared among *BRCA1* carriers vs matched wild‐type patients and among *BRCA2* carriers vs matched wild‐type patients. 88 *BRCA1* carriers and 75 *BRCA2* carriers were matched to 226 and 242 wild‐type patients, respectively. The proportions and timing of experiencing any grade or grade 3/4 cytopenias during chemotherapy were not significantly different for *BRCA1* carriers or *BRCA2* carriers vs matched wild‐type patients. Proportions requiring treatment modifications and time to first modification were also similar. Patterns of blood count fluctuations over time in mutation carriers mirrored those in wild‐type patients overall and by the most common regimens. Women with an inherited mutation in *BRCA1* or *BRCA2* experience similar frequency, severity, and timing of hematologic toxicities during curative intent breast cancer chemotherapy as matched wild‐type patients. Our findings suggest that *BRCA1* or *BRCA2* haploinsufficiency is sufficient for adequate bone marrow reserve in the face of short‐term repetitive hematopoietic stressors.

## Graphical abstract

In this multicenter, retrospective, matched cohort study, we investigated whether women with an inherited mutation in BRCA1 or BRCA2 experience more severe hematologic toxicities during curative intent chemotherapy for breast cancer than similar women without a mutation. We found that the frequency, severity, and timing of toxicities were similar in these two groups. This study is reassuring that women with an inherited mutation are able to tolerate the repetitive bone marrow stress of modern chemotherapy regimens with usual supportive care measures as well as those without a mutation.

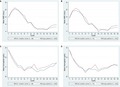

## 1 INTRODUCTION

Women who inherit a deleterious mutation in *BRCA1* or *BRCA2* are at increased risk of developing breast cancer. The cumulative breast cancer risk to age 80 is estimated at 72% and 69% for women with a *BRCA1* or *BRCA2* mutation, respectively.^1^ Prophylactic mastectomies and oophorectomies can substantially reduce this risk,^2^ but many women only learn of their mutation status at the time of a first cancer diagnosis or when a cancer occurs despite the preventative interventions. Thus, many will develop breast cancer which may require chemotherapy for optimal chance at cure.

Importantly, *BRCA1* and *BRCA2* are components of the Fanconi anemia (FA) DNA repair pathway,^3^ a key pathway involved in maintenance of normal hematopoietic stem cell function. Inheritance of two abnormal copies of *BRCA1* or *BRCA2* causes FA, an inherited bone marrow failure (BMF) syndrome characterized by increased sensitivity to DNA crosslinking agents, BMF, and a 785‐fold increased risk of developing hematologic malignancies.^4^, ^5^, ^6^, ^7^, ^8^ In contrast, inheriting one abnormal copy of *BRCA1* or *BRCA2* is more common, affecting 1 in 300 to 500 women in the general population,^9^ but the effect of a single mutation on bone marrow function is unclear.

Small studies have reported conflicting data as to whether women with a single inherited mutation in *BRCA1* or *BRCA2* experience excess hematologic toxicity during cytotoxic chemotherapy.^10^, ^11^, ^12^ Reported toxicities included more frequent dose‐limiting neutropenia and neutropenic fever, cases of therapy‐related leukemia, and lower baseline blood counts. However, conclusions were limited by small sample size, lack of a comparable control population, and/or inability to evaluate those with *BRCA1* vs *BRCA2* mutations separately.^10^, ^11^, ^12^, ^13^, ^14^ Thus, we performed a multicenter, retrospective, matched cohort study to compare the frequency, severity, and timing of hematologic toxicities occurring throughout the multicycle curative intent chemotherapeutic regimens for breast cancer in women with and without an inherited *BRCA1* or *BRCA2* mutation.

## 2 METHODS

### 2.1 Patient population

Women with localized breast cancer requiring chemotherapy were identified from cancer genetics clinic registries at the University of Chicago (UChicago; 1990‐present), NorthShore University HealthSystem (NorthShore; 1996‐present), University of Colorado Denver (UCD; 2002‐present), and Fox Chase Cancer Center (FCCC; 2000‐present). Eligibility required availability of complete chemotherapy administration records, complete blood count (CBC) data throughout chemotherapy, and results of prior *BRCA1* and *BRCA2* genetic testing. Those with metastatic disease, prior chemotherapy exposure, or who were pregnant were excluded. This study was approved by the Institutional Review Boards of all four centers in accordance with the Declaration of Helsinki.

### 2.2 Matching procedure

At each site, eligible women who tested positive for a deleterious *BRCA1* or *BRCA2* mutation (hereafter termed “mutation carriers”) were matched with women who tested negative (hereafter termed “wild‐type patients”) in bins based on the combination of age (less than age 50 years or 50+ years old), race (Caucasian, African American, or other), and planned chemotherapy regimen (exact) (Supporting Information 1). Using a random number generator, approximately one to four wild‐type patients were identified for each carrier in each bin. Together, all matched wild‐type patients and carriers in the same bin constituted a matching set. Clinical characteristics, chemotherapy administration doses and dates, CBC values prior to and following each cycle of chemotherapy, and supportive care measures, such as granulocyte colony‐stimulating factor (G‐CSF) administration and blood transfusions, were abstracted from medical records. Complications, including neutropenic fever, were recorded for each cycle. All toxicities were graded according to the Common Terminology Criteria for Adverse Events version 5^15^ (Supporting Information 2).

### 2.3 Study outcomes

For all comparisons, *BRCA1* and *BRCA2* mutation carriers were each compared to their own matched wild‐type patients. Wild‐type patients could be utilized in both the *BRCA1* and *BRCA2* cohort comparisons if they were matched in a set containing at least one *BRCA1* and one *BRCA2* mutation carrier. Primary outcomes were: (a) whether there was a difference in the proportion of patients developing any grade or severe (defined as grade 3/4) hematologic toxicity throughout the duration of the chemotherapy regimen; (b) whether the time to first severe hematologic toxicity differed; and (c) whether the change in specific blood count parameters over time with repeated cycles of chemotherapy differed between mutation carriers and matched wild‐type patients. Secondary outcomes included: whether treatment modifications or supportive care or baseline blood counts differed between mutation carriers and matched wild‐type patients.

### 2.4 Statistical analysis

Baseline clinical characteristics, blood count parameters, and proportions of patients experiencing specific hematologic toxicities or requiring specific dose modifications were compared between mutation carriers and matched wild‐type patients within each cohort using conditional logistic regression models (binary variables), multilevel mixed effects ordered logistic regression models (ordinal variables), multilevel multinomial logistic regression models (nominal variables), and multilevel mixed effects linear regression models (MEM; continuous variables). Observed dose modification‐free survival and severe hematologic toxicity‐free survival were calculated using the Kaplan‐Meier estimator, and overall differences were tested using the log rank test. Multilevel MEM were used to model patterns of log‐transformed white blood cell (WBC) count and hemoglobin over the duration of the chemotherapy regimen and test the effect of mutation status on each blood count parameter adjusted for relevant covariates, including age as a continuous variable. Final models for both WBC and hemoglobin were adjusted for time following cycle 1 (fixed effects and random effects), time following cycle 1 squared (fixed effects and random effects), treatment regimen, study site, and regimen by time interaction. To visualize patterns in blood count parameters, mean fitted values over time in mutation carriers vs matched wild‐type patients from the fully adjusted MEM models were plotted. Observed mean blood count values across all chemotherapy regimens and by specific regimens were also plotted. All tests of significance used a two‐sided *P* < .05. Statistical analyses were performed using Stata version 15 (StataCorp; College Station, TX).

## 3 RESULTS

### 3.1 Patient population

Among 5076 consented patients who underwent *BRCA1* and *BRCA2* genetic testing at one of the four sites, 1211 were eligible (Supporting Information 3) and 436 patients were matched successfully (Table 1). Among these, 436 patients were matched successfully, including 92 (16 *BRCA1*, 17 *BRCA2*, and 59 wild‐type) at UChicago, 203 (41 *BRCA1*, 29 *BRCA2*, 133 wild‐type) at NorthShore, 56 (12 *BRCA1*, 8 *BRCA2*, and 36 wild‐type) at UCD, and 85 (19 *BRCA1*, 21 B*RCA2*, 45 wild‐type) at FCCC. The combined *BRCA1* cohort consisted of 88 mutation carriers and 226 matched wild‐type patients (Supporting Information 4). The combined *BRCA2* cohort consisted of 75 mutation carriers and 242 matched wild‐type patients (Supporting Information 4).

**Table 1:** Patient, cancer, and treatment characteristics by cohort

	*BRCA1* mutation carriers (n = 88)	*BRCA1* matched wild‐type (n = 226)	*P*‐value	*BRCA2* mutation carriers (n = 75)	*BRCA2* matched wild‐type (n = 242)	*P*‐value
Age mean (SD)	44 (0.95)	46 (0.60)	**<.01**	44 (1.03)	46 (0.56)	.07
Race n (%)						
White	72 (82%)	186 (82%)	1.00	58 (77%)	201 (83%)	1.00
AA	7 (8%)	13 (6%)		9 (12%)	15 (6%)	
Other	9 (10%)	27 (12%)		8 (11%)	26 (11%)	
Breast cancer pathology						
Histology						
IDC	83 (94%)	194 (86%)		69 (92%)	209 (86%)	
ILC	0	19 (8%)	.99*	3 (4%)	18 (7%)	.35*
Other	5 (6%)	11 (5%)	.92*	3 (4%)	13 (5%)	.67*
Missing	0	2 (1%)		0	2 (1%)	
Grade						
I	0	16 (7%)	**<.01**	0	21 (9%)	**.01**
II	9 (10%)	90 (40%)		23 (31%)	90 (37%)	
III	78 (89%)	113 (50%)		51 (68%)	123 (51%)	
Missing	1 (1%)	7 (3%)		1 (1%)	8 (3%)	
Stage (n = 435^a^)						
1	33 (38%)	57 (25%)	**<.01**	21 (28%)	64 (26%)	.51
2	46 (52%)	109 (48%)		39 (52%)	122 (50%)	
3	8 (9%)	56 (25%)		15 (20%)	53 (22%)	
Missing	1 (2%)	4 (2%)		0	3 (1%)	
Receptor status						
ER+	21 (24%)	157 (69%)	**<.01**	54 (72%)	170 (70%)	.46
PR+	17 (19%)	132 (58%)	**<.01**	50 (67%)	148 (61%)	.05
HER2 amplified	5 (6%)	6 (3%)	.15	8 (11%)	11 (5%)	.57
Triple‐negative	65 (74%)	65 (29%)	**<.01**	16 (21%)	68 (28%)	.13
Treatment						
Surgery (n = 435^b^)						
Lumpectomy	29 (33%)	106 (47%)	**<.01** **	26 (35%)	108 (45%)	**<.01** **
Unilateral mastectomy	12 (14%)	63 (28%)	**<.01** **	15 (20%)	70 (29%)	**<.01** **
Bilateral mastectomy	47 (53%)	54 (24%)		33 (44%)	61 (25%)	
Missing	0	3 (1%)		1 (1%)	3 (1%)	
Chemotherapy regimen						
AC‐based regimen	73 (83%)	180 (80%)	1.00	54 (72%)	181 (75%)	1.00
TC	12 (14%)	41 (18%)		17 (23%)	54 (22%)	
Platinum‐based	3 (3%)	5 (2%)		3 (4%)	5 (2%)	
Other	0	0		1 (1%)	2 (1%)	
Additional treatment details						
Chemo given following surgery	67 (76%)	173 (77%)	.70	60 (80%)	184 (76%)	.52
Planned G‐CSF	78 (89%)	203 (90%)	.92	61 (81%)	215 (89%)	.75
Hormone blockade	23 (26%)	157 (69%)	**<.01**	52 (69%)	168 (69%)	.80
Radiation	36 (41%)	176 (78%)	**<.01**	39 (51%)	179 (74%)	**<.01**
Year of treatment, median (range)	2009 (2001‐2016)	2011 (1995‐2017)	**.01**	2012 (2000‐2015)	2011 (1995‐2017)	.30

Abbreviations: AA, African American; AC, anthracycline and cyclophosphamide; ER+, estrogen receptor‐positive; G‐CSF, granulocyte colony‐stimulating factor; IDC, invasive ductal carcinoma; ILC, invasive lobular carcinoma; PR+, progesterone receptor‐positive; SD, standard deviation; TC, taxotere cyclophosphamide.

a One patient with sternal invasion (Stage IV) was included due to lack of widely metastatic disease.

b One patient with occult primary underwent axillary lymph node dissection without breast surgery.

* *P*‐value for effect of BRCA status on being in ILC or other category vs IDC category.

** *P*‐value for effect of BRCA status on being in lumpectomy or unilateral mastectomy categories vs bilateral mastectomy category.

*P*‐value is bold if met statistical significance.

### 3.2 Breast cancer characteristics

As expected,^16^ compared to matched wild‐type patients, *BRCA1* mutation carriers more often developed higher grade (*P* < .01), estrogen receptor (ER)‐negative (*P* < .01), progesterone receptor‐negative (*P* < .01), and triple‐negative cancers (*P* < .01) (Table 1). In contrast, the majority of both *BRCA2* mutation carriers and their matched wild‐type patients developed ER+ breast cancers and had overall similar breast cancer characteristics, except that *BRCA2* mutation carriers more often developed higher grade cancers (*P* = .01).

### 3.3 Treatment characteristics

Mutation carriers underwent bilateral mastectomy more often than matched wild‐type patients (*P* < .01 for both *BRCA1* and *BRCA2* cohort comparisons) (Table 1). Approximately, 80% of mutation carriers and wild‐type patients in both cohorts were given adjuvant chemotherapy following surgery rather than prior to surgery. Year of treatment was similar for *BRCA2* mutation carriers vs wild‐type (median year of treatment 2012 vs 2011, *P* = .30), but was significantly earlier in those with a *BRCA1* mutation vs matched wild‐type patients (2009 vs 2011, *P* = .01).

In total, 3928 cycles of chemotherapy distributed among 15 regimens were given. Patients in the *BRCA1* and *BRCA2* cohort received 10 and 14 different regimens, respectively (Supporting Information 5). The majority in both cohorts received anthracycline and cyclophosphamide (AC)‐based regimens (83% vs 80% of *BRCA1* mutation carriers vs wild‐type; 72% vs 75% of *BRCA2* mutation carriers vs wild‐type) (Table 1). Dose‐dense AC followed by either dose‐dense paclitaxel (n = 33 and 73 *BRCA1* mutation carriers and wild‐type, respectively; n = 18 and 72 *BRCA2* mutation carriers and wild‐type, respectively) or weekly paclitaxel (n = 20 and 61 *BRCA1* mutation carriers and wild‐type, respectively; n = 15 and 62 *BRCA2* mutation carriers and wild‐type, respectively) were the two most common regimens. G‐CSF support was a planned part of the regimen in 89% and 90% of *BRCA1* mutation carriers and wild‐type patients (*P* = .92) and 81% and 89% of *BRCA2* mutation carriers and wild‐type patients (*P* = .75), respectively.

### 3.4 Baseline blood counts

**Figure 1:**
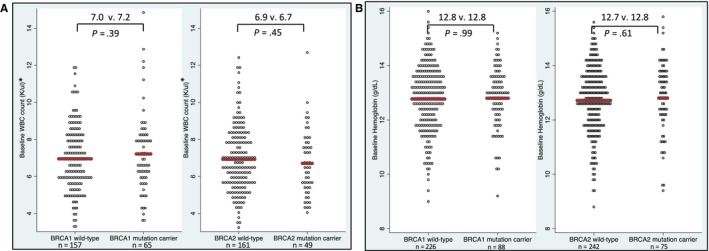
Baseline white blood count and hemoglobin values by cohort. *WBC excludes docetaxel based regimens due to pretreatment with corticosteroid that raises baseline WBC (see Figure S4). Abbreviations: WBC, white blood cell count (10 × 3/μL); Hgb, hemoglobin (g/dL)

Mean baseline blood count parameters were within the normal range and did not differ significantly between mutation carriers and wild‐type patients in either cohort (Figure 1 and Supporting Information 6 and 7).

### 3.5 Primary outcomes

#### 3.5.1 Proportion developing toxicities and timing of first severe toxicity

**Figure 2:**
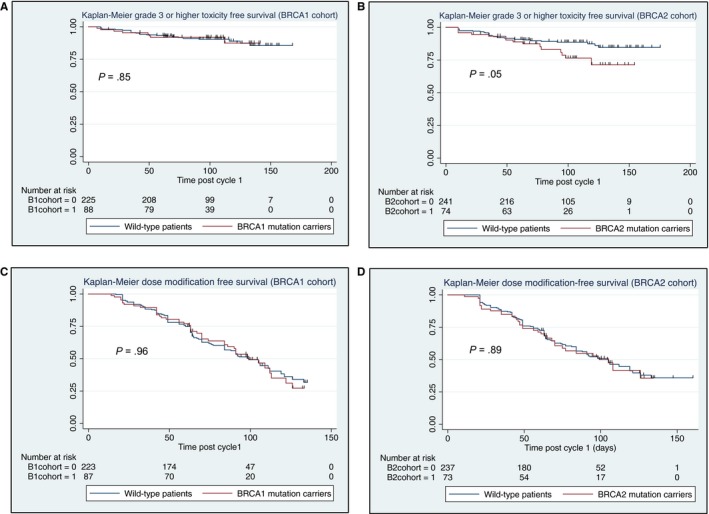
Grade 3 or higher hematologic toxicity‐free (A,B) and dose modification‐free (first dose reduction, dose delay, early chemotherapy cessation, extra granulocyte colony‐stimulating factor administration, or addition of erythropoietin support) survival (C,D) in days post start of cycle 1 by cohort: *BRCA1* (A,C) and *BRCA2* (B,D)

Nearly all patients in both cohorts developed at least grade 1 hematologic toxicity, with grade 1 or 2 hemoglobin decreases as the most common toxicities (Table 2). Grade 3 or higher toxicities were uncommon, occurring in <10% of patients in both cohorts. Leukopenia was the most common severe toxicity, occurring in 7% and 8% of *BRCA1* carriers and wild‐type (*P* = .58) and 15% and 10% of *BRCA2* carriers and wild‐type (*P* = .24), respectively. Overall, the proportion of patients who experienced any grade or grade 3 or higher toxicities did not differ between mutation carriers and wild‐type patients in either cohort (Table 2 and Supporting Information 8), with the exception of grade 1 or higher anemia, which occurred less often in mutation carriers in both cohorts (90% vs 96% (*P* = .05) and 80% vs 94% (*P* < .01) for *BRCA1* and *BRCA2* mutation carriers vs wild‐type patients, respectively). Observed timing of grade 3 or higher hematologic toxicities was similar in *BRCA1* carriers vs wild‐type patients (log rank *P* = .85), whereas there was a trend toward shorter time to first grade 3 or higher toxicity in *BRCA2* carriers as compared to wild‐type patients (log rank *P* = .05) (Figure 2A,B).

**Table 2:** Hematologic toxicities by grade and cohort

Toxicity n (%)	*BRCA1* mutation carriers (n = 88)	*BRCA1* matched wild‐type (n = 226)	OR (95% CI)	*P*‐value	*BRCA2 *mutation carriers (n = 75)	*BRCA2* matched wild‐type (n = 242)	OR (95% CI)	*P*‐value
Hemoglobin decreased (anemia)								
Any grade	79 (90%)	216 (96%)	0.34 (0.13‐0.99)	.05	60 (80%)	227 (94%)	0.27 (0.11‐0.70)	**<.01**
Grade 3/4^a^	4 (5%)	8 (4%)	1.10 (0.32‐3.80)	.88	7 (9%)	12 (5%)	1.90 (0.71‐5.07)	.20
RBC transfusion	4 (5%)	7 (3%)	1.21 (0.34‐4.29)	.77	7 (9%)	9 (4%)	2.71 (0.94‐7.77)	.06
WBC count decreased								
Any grade	39 (44%)	116 (51%)	0.64 (0.36‐1.15)	.14	41 (55%)	130 (54%)	0.85 (0.47‐1.54)	.59
Grade 3/4	6 (7%)	18 (8%)	0.76 (0.29‐1.99)	.58	11 (15%)	23 (10%)	1.63 (0.73‐3.67)	.24
Neutropenic fever	5 (6%)	11 (5%)	1.06 (0.35‐3.19)	.91	6 (8%)	18 (7%)	0.88 (0.32‐2.44)	.81
Platelet count decreased								
Any grade	9 (10%)	32 (14%)	0.70 (0.31‐1.58)	.39	11 (15%)	35 (14%)	1.10 (0.49‐2.46)	.83
Grade 3/4	0	1 (0.4%)		1.00	1 (1%)	1 (0.4%)	2.24 (0.11‐44.88)	.60

Abbreviations: RBC, red blood cell transfusion; WBC, white blood cell.

a Includes need for red blood cell transfusion (grade 3 toxicity).

*P*‐value is bold if met statistical significance.

#### 3.5.2 Timing and frequency of dose modifications

Median time to first chemotherapy dose modification was similar for mutation carriers and wild‐type patients in both cohorts (98 vs 98 days, log rank *P* = .96 for *BRCA1* carriers vs wild‐type, and 102 vs 104 days, log rank *P* = .89 for *BRCA2* carriers vs wild‐type, respectively) (Figure 2C,D). In total, a treatment modification, including chemotherapy dose delay, dose reduction, omitting planned cycles, administration of G‐CSF beyond preplanned doses, and/or addition of erythropoietin support, was required in 52% and 50% of *BRCA1* mutation carriers and wild‐type patients (*P* = .76) and in 49% and 50% of *BRCA2* mutation carriers and wild‐type patients (*P* = .96), respectively. Proportions requiring specific treatment modifications did not differ significantly by mutation status in either cohort (Supporting Information 9).

#### 3.5.3 Change in blood count parameters over time

**Figure 3:**
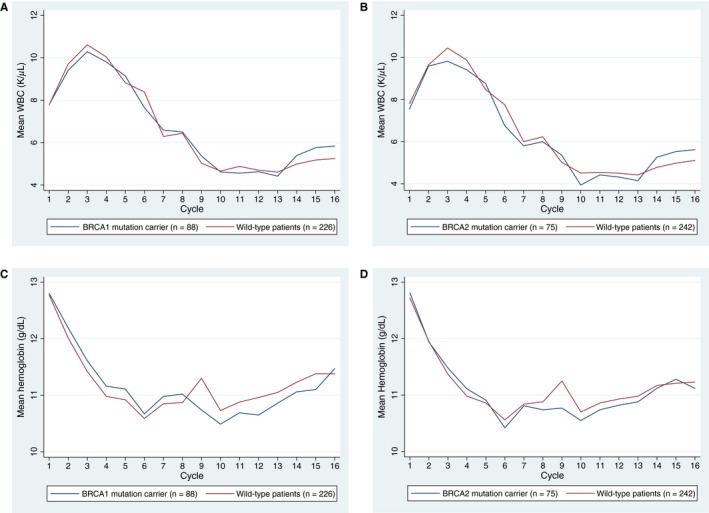
Observed mean white blood cell count (WBC) and hemoglobin values at time of each chemotherapy cycle. *BRCA1* mutation carrier versus wild‐type patients’ mean A. WBC (K/μL) and C. hemoglobin (g/dL). *BRCA2* mutation carrier versus wild‐type patients’ mean B. WBC (K/μL) and D. hemoglobin (g/dL)

Patterns of blood count fluctuations varied with time (Figure 3 and Supporting Information 10‐12). Using the fully adjusted two level MEM model, we did not find a significant difference in WBC (relative difference for *BRCA1* carriers vs wild‐type 1% [95% CI −5 to 8; *P* = .70]; relative difference for *BRCA2* carriers vs wild‐type 1% [95% CI −8 to 7, *P* = .88]) or hemoglobin over time (relative difference for *BRCA1* carriers vs wild‐type 1% [95% CI −0.3 to 3; *P* = .12]; relative difference for *BRCA2* carriers vs wild‐type 0.05% [95% CI −2 to 2; *P* = .65]). There was no significant difference in *BRCA* status by regimen, time, or time squared interaction.

Blood count patterns differed by chemotherapy regimen and within regimen by drug and dose intensity (Supporting Information 13‐14). Patterns in mutation carriers closely followed those in wild‐type patients. For example, weekly paclitaxel more frequently lowered the WBC count below baseline as compared to dose‐dense paclitaxel, but patterns in mutation carriers closely followed those in matched wild‐type patients.

## 4 DISCUSSION

Combining cases from four centers, we show that the overall proportions of *BRCA1* and *BRCA2* mutation carriers who develop mild as well as severe hematologic toxicities during chemotherapy for localized breast cancer are similar to matched patients who do not carry a mutation. Patterns of blood count fluctuations over time overall and within the regimens, most commonly used in current clinical practice, were also similar. Furthermore, there was no significant difference in treatment modification proportions nor timing of first modification during curative intent chemotherapy between *BRCA1* and *BRCA2* mutation carriers or matched wild‐type patients.

From these data, short‐term hematologic toxicities during breast cancer chemotherapy appear similar which should be reassuring for clinicians administering, and patients with a *BRCA1* or *BRCA2* mutation receiving, chemotherapy. However, whether the DNA damage that results from chemotherapy exposures is repaired by similar mechanisms and/or to a similar extent in individuals who carry *BRCA1* and *BRCA2* mutations vs patients without a mutation remains unknown. If there are differences in the integrity of DNA repair at the hematopoietic stem cell level, this could impact longer term hematopoiesis and myeloid malignancy risk. To date, few cases of therapy‐related leukemia have been reported in long‐term follow‐up studies of *BRCA1* and *BRCA2* mutation carriers, but these studies have been limited by significant loss to follow‐up, a critical problem for identifying a toxicity that can occur up to 10 years later.^17^, ^18^, ^19^ Thus, longer term hematologic toxicity differences remain an open question and require further study.

We observed single individuals with greater than expected hematologic toxicities. Although not examined in this study, others have recently observed an increased need for transfusions and lower blood counts among individuals receiving chemotherapy who have evidence of clonal hematopoiesis (CH) in their blood.^20^ Given that CH is also a risk factor for future hematologic malignancy development,^21^ the incidence of this finding at baseline as well as following chemotherapy in *BRCA1* and *BRCA2* carriers vs individuals without a mutation during chemotherapy warrants further investigation. Most importantly, patients with greater than expected toxicity should be evaluated for alternative etiologies.

Our study has limitations. First, our study is enriched for women with young age at diagnosis, triple‐negative breast cancer, or a strong family history. Second, women enrolled in this study underwent varying genetic testing ranging from *BRCA1* and *BRCA2* Ashkenazi Jewish founder mutation analysis only to multigene panel testing. Thus, the “wild‐type” patients may be enhanced for inherited breast cancer gene mutations that may also affect DNA repair, which could bias these results toward the finding of no difference. Third, despite recruiting from four high volume centers, sample sizes for many individual regimens were still relatively small and regimen‐specific toxicity differences could have been missed. Nonetheless, the regimens most widely used in practice were given to the majority of patients in this study. Given the many different chemotherapy regimens in routine use for breast cancer patients, detection of germline genotype by regimen‐specific toxicities will require incorporating longitudinal toxicity studies into large prospective clinical treatment trials that include modern comprehensive germline genetic testing for all patients. These studies will be especially relevant for trials incorporating poly ADP ribose polymerase inhibitors, a drug class of specific interest due to its preferential use in *BRCA1* and *BRCA2* mutation carriers, known dose‐limiting hematologic toxicities, and association with therapy‐related myeloid neoplasms.^22^, ^23^ Lastly, given that G‐CSF support is a required component for safe delivery of many modern breast cancer regimens, we were unable to rule out differences in toxicities by mutation status in the absence of this agent, but this is likely not clinically relevant.

## 5 CONCLUSIONS

Women carrying an inherited *BRCA1* or *BRCA2* mutation experience similar severity, frequency, and timing of hematologic toxicity as women without a germline mutation while receiving modern day chemotherapy and supportive care for localized breast cancer. These data suggest that haploinsufficiency of *BRCA1* or *BRCA2* is sufficient for adequate bone marrow reserve in the face of short‐term repeated hematopoietic stressors.

## CONFLICT OF INTEREST

Olufunmilayo Olopade is cofounder of CancerIQ.

## AUTHOR CONTRIBUTIONS

AHW study conceptualization, data curation, formal data analysis, funding acquisition, writing of original draft, review and editing; HK data curation. review and editing; JD data curation, review and editing; DH formal data analysis, investigation and methodology, review and editing; EAH formal data analysis, investigation and methodology, review and editing; SMN data curation, review and editing; LCB data curation, review and editing; LGG data curation, review and editing; Heather Willems: data curation, review and editing; Mary B. Daly: resources, review and editing; AA data curation, resources, review and editing; OIO resources, review and editing; PJH study conceptualization, data curation, resources, review and editing; ES study conceptualization, data curation, resources, supervision, review and editing; DH study conceptualization, formal data analysis, investigation and methodology, funding acquisition, supervision, review and editing; EO: study conceptualization, data curation, formal data analysis, funding acquisition, supervision, writing of original draft, review and editing; JEC study conceptualization, data curation, formal data analysis, funding acquisition, investigation and methodology, supervision, writing of original draft, review and editing.

## Data Availability

The data that support the findings of this study are available on request from the corresponding author. The data are not publicly available due to privacy or ethical restrictions.
