# Supplementary material for: Hematologic toxicity in BRCA1 and BRCA2 mutation carriers during chemotherapy: A retrospective matched cohort study
Source: Cancer Med. 2019 Aug 12;8(12):5609–18. doi: 10.1002/cam4.2471 (PMC6745828; doi:10.1002/cam4.2471)
Supplement: Supplementary file 1 [file CAM4-8-5609-s001.docx]

**Supporting Information and Methods**

**Supplement 1.** Retrospective matched cohort study design diagram

**Supplement 2.** Common Terminology Criteria for Adverse Events version 5^1^ Hematologic Toxicity Grading Criteria

| **Hematologic Parameter** | **Grade 1** | **Grade 2** | **Grade 3** | **Grade 4** | **Grade 5** |
| --- | --- | --- | --- | --- | --- |
| White blood cell count decreased | <LLN –3000/mm^3^ | 3000 –2000/mm^3^ | 2000 –1000/mm^3^ | <1000/mm^3^ | _ |
| Absolute neutropil count decreased | <LLN –1500/mm^3^ | 1500 –1000/mm^3^ | 1000 – 500/mm^3^ | <500/mm^3^ | _ |
| Anemia  (Hemoglobin decreased) | <LLN–10.0 g/dL | 10.0 –8.0 g/dL | <8.0 g/dL or transfusion indicated | Life threatening; urgent intervention indicated | Death |
| Platelet count decreased | <LLN – 75,000/mm^3^ | 75,000 – 50,000/mm^3^ | 50,000 – 25,000/mm^3^ | <25,000/mm^3^ | _ |
| Febrile neutropenia | _ | _ | ANC <1000/mm^3^ with single temperature >38.3 C or >/= 38 C for more than 1 hour | Life threatening; urgent intervention indicated | Death |
| Toxicity Grading Key: grade 1 = mild; grade 2 = moderate; grade 3 = severe or medically significant but not immediately life-threating; grade 4 = life-threatening consequences; grade 5 = death related to adverse event | | | |  |  |

**Supplement 3.** Consort Diagram


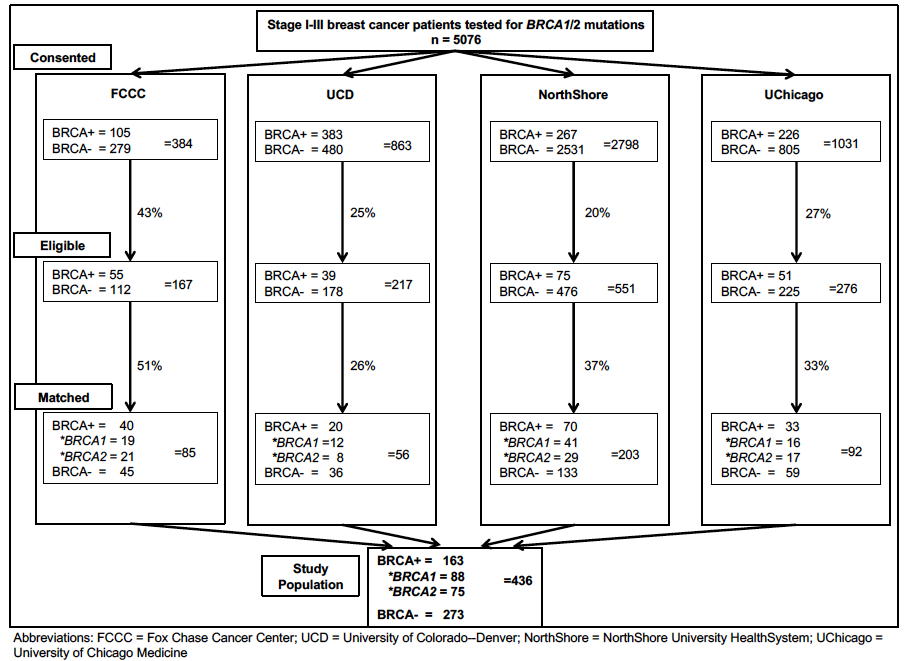


| **Supplement 4.** Specific pathogenic *BRCA1* and *BRCA2* variants found in the mutation carriers in this study | | | |
| --- | --- | --- | --- |
| **Gene** | **cDNA*** | **Protein** | **Frequency** |
| ***BRCA1*** |  |  |  |
|  | c.66dupA | p.Glu23Argfs | 2 |
|  | c.68_69delAG | p.Glu23Valfs | 25 |
|  | c.181T>G | p.Cys61Gly | 2 |
|  | c.191G>A | p.Cys64Tyr | 1 |
|  | c.213-11T>G |  | 1 |
|  | c.303T>A | p.Tyr101Ter | 1 |
|  | c.815_824dupAGCCATGTGG | p.Thr276Alafs | 2 |
|  | c.1105_1106insTC | p.Asp369Valfs | 1 |
|  | c.1175_1214del40 | p.Leu392Glnfs | 1 |
|  | c.1556delA | p.Lys519Argfs | 1 |
|  | c.1687C>T | p.Gln563Ter | 1 |
|  | c.1892dupT | p.Ser632Lysfs | 1 |
|  | c.1916T>A | p.Leu639Ter | 1 |
|  | c.2457delC | p.Asp821Ilefs | 1 |
|  | c.2475delC | p.Asp825Glufs | 1 |
|  | c.2560_2561dupGC | p.Gln855Leufs | 1 |
|  | c.2681_2682delAA | c.2681_2682delAA | 1 |
|  | c.2722G>T | p.Glu908Ter | 1 |
|  | c.2866_2870delTCTCA | p.Ser956Valfs | 1 |
|  | c.2934G>T | p.Tyr978Ter | 1 |
|  | c.3018_3021delTTCA | p.His1006Glnfs | 1 |
|  | c.3108dupT | p.Lys1037Terfs | 1 |
|  | c.3138_3141delAGTA | p.Gly1048ProfsX13 | 1 |
|  | c.3228_3229delAG | p.Gly1077Alafs | 1 |
|  | c.3607C>T | p.Arg1203Ter | 1 |
|  | c.3756_3759delGTCT | p.Ser1253Argfs | 2 |
|  | c.4035delA | p.Glu1346Lysfs | 1 |
|  | c.4065_4068delTCAA | p.Asn1355Lysfs | 1 |
|  | c.4357+1G>A |  | 2 |
|  | c.4391_4393delCTAinsTT | p.Pro1464Leufs | 1 |
|  | c.5068A>T | p.Lys1690Ter | 1 |
|  | c.5075-1G>A |  | 1 |
|  | c.5102_5103delTG | p.Leu1701GInsfs | 1 |
|  | c.5137delG | p.Val1713Terfs | 1 |
|  | c.5251C>T | p.Arg1751Ter | 2 |
|  | c.5266dupC | p.Gln1756Profs | 7 |
|  | c.5468-1G>A |  | 1 |
|  | c.4936delG | p.val1646Serfs | 1 |
|  | c.4054G>T | p.Glu1352Ter | 1 |
|  | c.4185+1G>T |  | 1 |
|  | c.4986+3G>C |  | 1 |
|  | c.4986+6T>C |  | 1 |
|  | c.5096G>A | p.Arg1699Gin | 1 |
|  | c.5468-2A>G |  | 1 |
|  | del ex3 |  | 1 |
|  | del ex11-12 |  | 1 |
|  | del 12-14 |  | 1 |
|  | del ex14-20 |  | 1 |
|  | del ex16-18 |  | 1 |
|  | del ex17 |  | 1 |
|  | del ex20 |  | 1 |
|  | del ex22 |  | 1 |
|  |  | **Total** | **88** |
| ***BRCA2*** |  |  |  |
|  | c.658_659delGT | p.Val220Ilefs | 3 |
|  | c.771_775delTCAAA | p.Asn257Lysfs | 1 |
|  | c.1265delA | p.Asn422Ilefs | 1 |
|  | c.1552delG | p.Ala518GInfs | 1 |
|  | c.1813dupA | p.Ile605Asnfs | 4 |
|  | c.1832C>G | p.Ser611Ter | 1 |
|  | c.2224C>T | p.Gln742Ter | 1 |
|  | c.2808_2811del | p.Ala938Profs | 3 |
|  | c.3170_3174delAGAAA | p.Lys1057Thrfs | 1 |
|  | c.3680_3681delTG | p.Leu1227Glnfs | 1 |
|  | c.3820_3823delAAGA | p.Lys1274Terfs | 1 |
|  | c.3847_3848delGT | p.Val1283Lysfs | 1 |
|  | c.3860delA | p.Asn1287Ilefs | 1 |
|  | c.3967A>T | p.Lys1323Ter | 1 |
|  | c.4163_4164delCTinsA | p.Thr1388Asnfs | 1 |
|  | c.4638delT | p.Phe1546Leufs | 1 |
|  | c.4943delC | p.Ala1648fs | 1 |
|  | c.5130_5133delTGTA | p.Tyr1710Terfs | 1 |
|  | c.5238dupT | p.Asn1747Terfs | 1 |
|  | c.5277+1G>A |  | 1 |
|  | c.5645C>A | p.Ser1882Ter | 1 |
|  | c.5682C>G | p.Tyr1894Ter | 2 |
|  | c.5722_5723delCT | p.Leu1908Argfs | 1 |
|  | c.5864C>A | p.Ser1955Ter | 1 |
|  | c.5946delT | p.Ser1982Argfs | 16 |
|  | c.6267_6269delGCAinsC | p.Glu2089Aspfs | 1 |
|  | c.6405_6409delCTTAA | p.Asn2135Lysfs | 1 |
|  | c.6444dupT | p.Ile2149Tyrfs | 1 |
|  | c.6468_6469delTC | p.Gln2157Ilefs | 1 |
|  | c.6566dupA | p.Asn2189Lysfs | 1 |
|  | c.6757_6758delCT | p.Leu2253Phefs | 1 |
|  | c.6859_6863delAGAAA | p.Arg2287Leufs | 1 |
|  | c.7024C>T | p.Gln234Ter | 1 |
|  | c.7258G>T | p.Glu2420Ter | 1 |
|  | c.7366C>T | p.Gln2456Ter | 1 |
|  | c.7480C>T | p.Arg2494Ter | 1 |
|  | c.7558C>T | p.Arg2520Ter | 1 |
|  | c.8165C>G | p.Thr2722Arg | 1 |
|  | c.8168A>G | p.Asp2723Gly | 1 |
|  | c.8247_8248delGA | p.Lys2750Aspfs | 1 |
|  | c.8297delC | p.Thr2766Asnfs | 3 |
|  | c.8400_8402delTTTins4 |  | 1 |
|  | c.8754+5G>A |  | 1 |
|  | c.8953+1G>T |  | 1 |
|  | c.9117G>A | p.Pro3039= | 1 |
|  | c.9154C>T | p.Arg3052Trp | 1 |
|  | c.9253dupA | p.Thr3085Asnfs | 1 |
|  | c.9329insA | p.Asp3110fs | 1 |
|  | c.9382C>T | p.Arg3128Ter | 1 |
|  | del ex1-2 |  | 1 |
|  |  | **Total** | **75** |
| **BRCA1* NM 007294.3, *BRCA2* NM_000059.3 | | | |

**Supplement 5.** Chemotherapy Regimen Treatment and Complete Blood Count Measurement Schema

**Supplement 6.** Baseline mean corpuscular volume (A.), red cell distribution width (B.), and platelet count (C.) by cohort

**Supplement 7**. Baseline white blood cell count and absolute neutrophil count including (A & B) or excluding (C & D) docetaxol-based regimens which include steroid premedication 24 hours prior to chemotherapy administration by cohort

**Supplement 8.** Two level mixed effects ordered logistic regression random intercept models of the effect of carrying an inherited mutation in *BRCA1* or *BRCA2* versus not carrying a mutation on maximum hematologic toxicity grade level during the entire course of chemotherapy adjusted for matching bin by study cohort

|  | **Covariate of interest & effect estimates** | | | |
| --- | --- | --- | --- | --- |
|  | ***BRCA1* mutation carrier vs. non-mutation carrier status^#^** |  | ***BRCA2* mutation carrier vs. non-mutation carrier status^^^** |  |
| **Outcome** | **Odds Ratio**  **(95% CI)** | **p-value** | **Odds Ratio**  **(95% CI)** | **p-value** |
| **Maximum hemoglobin toxicity**** | 0.85  (0.50 to 1.45) | 0.56 | 0.91  (0.52 to 1.59) | 0.74 |
| **Maximum WBC count toxicity**** | 0.76  (0.46 to 1.24) | 0.27 | 0.98  (0.59 to 1.65) | 0.95 |
| **Maximum hematologic toxicity**^$^** | 0.66  (0.41 to 1.08) | 0.10 | 0.84  (0.50 to 1.43) | 0.53 |

**Toxicity grade categories: 0, 1 or higher, 2 or higher, 3 or higher

^$^Composite of maximum white blood cell, red blood cell and platelet toxicities

^#^*BRCA1* cohort (n=88 *BRCA1* mutation carriers and n=226 matched wild-type patients)

^^^*BRCA2* cohort (n=75 *BRCA1* mutation carriers and n=242 matched wild-type patients)

| **Supplement 9.** Frequency and type of chemotherapy regimen dose modifications by cohort | | | | | | | | | | |  | |  | |  |
| --- | --- | --- | --- | --- | --- | --- | --- | --- | --- | --- | --- | --- | --- | --- | --- |
|  | ***BRCA1* mutation carriers  (n=88)** | ***BRCA1* matched wild-type (n=226)** | **OR**  **(95% CI)** | ***p*-value** | ***BRCA2* mutation carriers  (n=75)** | ***BRCA2* matched wild-type (n=242)** | **OR**  **(95% CI)** | ***p*-value** |  |  |  |  |  |  |  |
| **Specific Modifications, n (%)** |  |  |  |  |  |  |  |  |  |  |  |  |  |  |  |
| Dose delay | 23 (26%) | 79 (35%) | 0.57  (0.31-1.06) | 0.08 | 25 (33%) | 78 (32%) | 1.16  (0.59-2.03) | 0.67 |  |  |  |  |  |  |  |
| Dose reduction | 15 (17%) | 33 (15%) | 1.33  (0.65-2.69) | 0.43 | 9 (12%) | 37 (15%) | 0.79  (0.34-1.84) | 0.59 |  |  |  |  |  |  |  |
| Early cessation | 7 (8%) | 27 (12%) | 0.67  (0.28-1.59) | 0.36 | 9 (12%) | 32 (13%) | 0.78  (0.34-1.83) | 0.57 |  |  |  |  |  |  |  |
| Unplanned G-CSF | 8 (9%) | 13 (6%) | 1.41  (0.56-3.51) | 0.47 | 11 (15%) | 21 (9%) | 1.45  (0.64-3.32) | 0.37 |  |  |  |  |  |  |  |
| Erythropoietin support | 6 (7%) | 13 (6%) | 1.20  (0.42-3.42) | 0.73 | 4 (5%) | 16 (7%) | 0.57  (0.12-2.76) | 0.48 |  |  |  |  |  |  |  |
| **Total Cycles Planned,**  **n (mean per person)** | **778 (8.8)** | **2042 (9.0)** |  |  | **664 (8.9)** | **2230 (9.2)** |  |  |  |  |  |  |  |  |  |
| **Total Cycles Omitted,**  **n (% of total planned)** | **36 (5)** | **66 (3)** |  |  | **47 (7)** | **80 (4)** |  |  |  |  |  |  |  |  |  |
| Abbreviations: G-CSF = granulocyte colony stimulating factor | | | | | | | | | |  | |  | |  | |

**Supplement 10.** Two level mixed effects linear regression adjusting for BRCA status (0/1), Time following Cycle 1 (months, continuous, fixed effects and random effects), Time following Cycle 1 squared (fixed effects and random effects), Treatment Regimen, Site, and Regimen by Time interaction was used to obtain fitted mean log transformed white blood cell count and hemoglobin values at time of each chemotherapy cycle by cohort. *BRCA1* mutation carrier versus wild-type patients’ mean WBC (A) and hemoglobin (C). *BRCA2* mutation carrier versus wild-type patients’ mean WBC (B) and hemoglobin (D)

**Supplement 11**. Regimen Specific *BRCA1* cohort observed mean white blood cell count and hemoglobin values at time of each chemotherapy cycle standardized to entire cohort mean baseline values. **A, C.** WBC and hemoglobin values during dose dense doxorubicin plus cyclophosphamide followed by weekly paclitaxel. **B, D.** WBC and hemoglobin values during dose dense doxorubicin plus cyclophosphamide followed by dose dense paclitaxel

**A.**

**D.**

**C.**

**B.**

**Supplement 12**. Regimen Specific *BRCA2* cohort observed mean white blood cell count and hemoglobin values at time of each chemotherapy cycle standardized to entire cohort mean baseline values. **A, C.** WBC and hemoglobin values during dose dense doxorubicin plus cyclophosphamide followed by weekly paclitaxel. **B, D.** WBC and hemoglobin values during dose dense doxorubicin plus cyclophosphamide followed by dose dense paclitaxel

**A.**

**D.**

**C.**

**B.**

**Supplement 13**. Absolute neutrophil count changes over time in individual *BRCA1* mutation carriers getting dose dense doxorubicin and cyclophosphamide (DDAC) followed by weekly paclitaxel (weekly T) (A.) or DDAC followed by dose dense paclitaxel (DDT) (B.) versus similarly treated matched wild-type patients (C. and D., respectively). The red line is at 1 K/uL, the lower limit of grade 2 toxicity

**Supplement 14.** Hemoglobin changes over time in individual *BRCA1* mutation carriers getting dose dense doxorubicin and cyclophosphamide (DDAC) followed by dose dense paclitaxel (DDT) (A.) or DDAC followed by weekly paclitaxel (weekly T) (B.) versus similarly treated matched wild-type patients (C. and D., respectively). The red line is at 8 g/dL, the lower limit of grade 2 toxicity

**Supplemental Methods**

**Study Design**

The minimum sample size for this study was determined based on the observed 22% difference in the primary outcome of the proportion of patients developing any grade hematologic toxicity in Huzno et al.^2^ With a two sided α=0.05, a sample size of n=67 *BRCA1* mutation carriers, n=67 *BRCA2* mutation carriers, and each with n=67 matched wild-type patients would have 84% power to detect this expected difference. All eligible, matched mutation carriers beyond the minimum sample size were included to increase the power to detect smaller differences.

**References**

1. Common Terminology Criteria for Adverse Events (CTCAE), version 5.0. November 27, 2017.

2. Huszno J, Budryk M, Kolosza Z, et al: The influence of BRCA1/BRCA2 mutations on toxicity related to chemotherapy and radiotherapy in early breast cancer patients. *Oncology*, 2013; 85:278-82.
